# 

**Published:** 1856-10

**Authors:** 


### CONTENTS.

### ORIGINAL COMMUNICATIONS.

PAGE

ARTICLE I.—An Address before the Graduating Class of the Atlanta Medical College. By Hon. JOHN L. HARRIS of Atlanta, Ga.,........ 65

ARTICLE II.—Operation for Hare-Lip. By ISHAM H. RAGAN, M. D. of Lithonia, Ga.,........ 72

ARTICLE III.—Pompion Seed as a Tæniafuge. By JOHN W. JONES, M. D. of Atlanta, Georgia,........ 73

ARTICLE IV.—On Small Pox. By N. F. POWERS, M. D.,........ 76

**SELECTIONS.**

An Essay upon the Relation of Bilious and Yellow Fever,........ 82
Hints to Diagnosis,........ 96
Politics and Medicine,........ 102
Remarks on the Treatment of Intermittent Fever,........ 105
Extraction of Needles, &c., from the Human Body,........ 108
Laceration of the Cervix Uteri,........ 110
Observations on Indigenous Medical Plants,........ 112
Stricture of the Rectum,........ 113
Iodine as an Emmanagogue,........ 114

**EDITORIAL AND MISCELLANEOUS.**

Third Course of Lectures,........ 115
To our Patrons,........ 116
Essays on the Physiology of the Nervous System,........ 116
On Lactate of Zinc in Epilepsy,........ 122
The Abortion of Trade,........ 123
Regimen,........ 124
Cure of Itch in Half an Hour,........ 124
Glycerine as a substitute for Cod-liver Oil in Phthisis,........ 125
On Cantharidian and its Relation to Spanish Flies,........ 126
Influence of the Sun's Rays in Consumption,........ 126
Draper's Physiology,........ 128
Subscriptions Received,........ 128

